# Indoor Skydiving: An Emerging Cause of Anterior Shoulder Dislocations

**DOI:** 10.31486/toj.21.0027

**Published:** 2022

**Authors:** Nicholas L. Newcomb, David R. Lester, Lucas K. Keyt, Daniel M. Zumsteg, Piers A. Barry

**Affiliations:** ^1^ The University of Queensland Faculty of Medicine, Ochsner Clinical School, New Orleans, LA; ^2^ Virginia Commonwealth University School of Medicine, Richmond, VA; ^3^ University of California San Diego School of Medicine, La Jolla, CA; ^4^ Piers Barry MD Inc, San Francisco, CA

**Keywords:** *Athletic injuries*, *Bankart lesions*, *labral injuries*, *shoulder dislocation*

## Abstract

**Background:** The risks of indoor skydiving have not been extensively studied. Indoor skydiving facilities are often used for corporate events and parties and by relatively inexperienced participants who may not appreciate the risks involved. The abducted and externally rotated shoulder position, combined with nearby walls, tight spaces, and the strong airstream, has resulted in a pattern of shoulder dislocation injuries.

**Case Report:** A 26-year-old male presented with recurrent left shoulder instability after developing an engaging Hill-Sachs lesion following traumatic anterior shoulder dislocation while indoor skydiving. He entered the wind tunnel with his arms abducted and externally rotated. The wind created an upward force that held his arms in this position. As he reached with his left arm for the side of the tunnel to exit, his arm was forced into further external rotation, dislocating the shoulder. The patient was treated arthroscopically with a remplissage procedure and repair of the glenoid labrum. Postoperatively, he resumed his active lifestyle and sports without further dislocations or instability.

**Conclusion:** Indoor skydiving may pose a high risk of anterior dislocation because the shoulder is forced into abduction and external rotation in the free-fall position. We advise caution before participation in indoor skydiving by any individual, but especially those with a history of shoulder instability.

## INTRODUCTION

Patients characteristically at risk for anterior shoulder dislocation tend to be males aged 18 to 29 years who participate in contact sports or undergo military training.^1-3^ Anterior shoulder dislocations also occur in association with the sport of skydiving and are often attributed to the airstream force on the arm. In skydiving, the arms are abducted and externally rotated, and the airstream pushes the arms into further external rotation.^4,5^ This position aligns with the accepted mechanism of anterior shoulder dislocation.^6^

Skydiving has evolved into an indoor version in which a powerful fan blows air upwards to suspend the participant in an enclosed column. Advocates have pushed to make indoor skydiving an Olympic event.^7^ However, indoor skydiving facilities are often used for corporate events and parties and by relatively inexperienced participants who may not appreciate the risks involved. The abducted and externally rotated shoulder position, combined with nearby walls, tight spaces, and the strong airstream, has resulted in a pattern of shoulder dislocation injuries. Rodrigues reported that 61 of 120 (51%) injuries at a skydiving company occurred to the shoulder and ranged from pain to dislocations.^8^

We present a case of anterior shoulder dislocation that occurred during indoor skydiving.

## CASE REPORT

**Figure 1.**
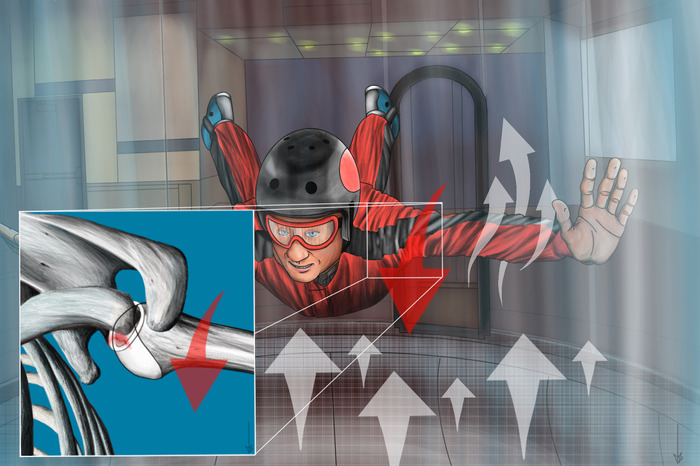
**Illustration demonstrating the mechanism of shoulder dislocation. The patient is in the wind tunnel with his arms abducted and externally rotated, with the wind pushing his arms into further external rotation. When he reached for the side of the wind tunnel while attempting to exit, his left arm was forced into further external rotation, causing an anterior dislocation of the left shoulder.** (Original art by Daniel M. Zumsteg, MD. Published with permission.)

A right-handed, 26-year-old male presented with recurrent left shoulder dislocations resulting in severe shoulder instability. He had initially dislocated his shoulder while indoor skydiving approximately 2 years prior to this consultation. He entered the wind tunnel with his arms abducted and externally rotated, and the airstream created an upward force that held his arms in position. While attempting to exit, he reached for the side of the tunnel with his left arm, and the arm was forced into further external rotation by the airstream. The patient felt his shoulder dislocate and then spontaneously relocate seconds later (Figure 1). He had had no shoulder injuries prior to the initial dislocation. After the dislocation, the patient completed extensive nonoperative rehabilitation that involved several months of physical therapy at the direction of an emergency department physician. Approximately 1 year later, the patient dislocated his shoulder a second time when he collided with another player while playing soccer. He again pursued physical therapy; however, his shoulder continued to dislocate with increasing frequency and minimal force, including during sleep.

Clinically, the patient's left shoulder was highly unstable, with apprehension at 60° of forward flexion and 30° of external rotation. He had a positive Jobe relocation test and sulcus sign, both indicating shoulder instability. The patient had no signs of systemic ligamentous laxity at the elbow or thumb. With a stabilizing posterior push, he exhibited bilateral shoulder flexion to 175° (normal flexion, 165°) and external rotation in full adduction to 95° bilaterally (normal rotation, 90°). He was tender over the left proximal biceps. He had mild pain and weakness with the empty can test, Speed test, and O’Brien test, indicating possible superior rotator cuff injury, injury to the long head of the biceps or superior glenoid labrum, and glenoid labrum injury, respectively. The lift-off test was normal, indicating an intact subscapularis. Sensory and vascular examinations were normal. The patient's medical history was negative for medications, tobacco, alcohol, and recreational drug use.

**Figure 2.**
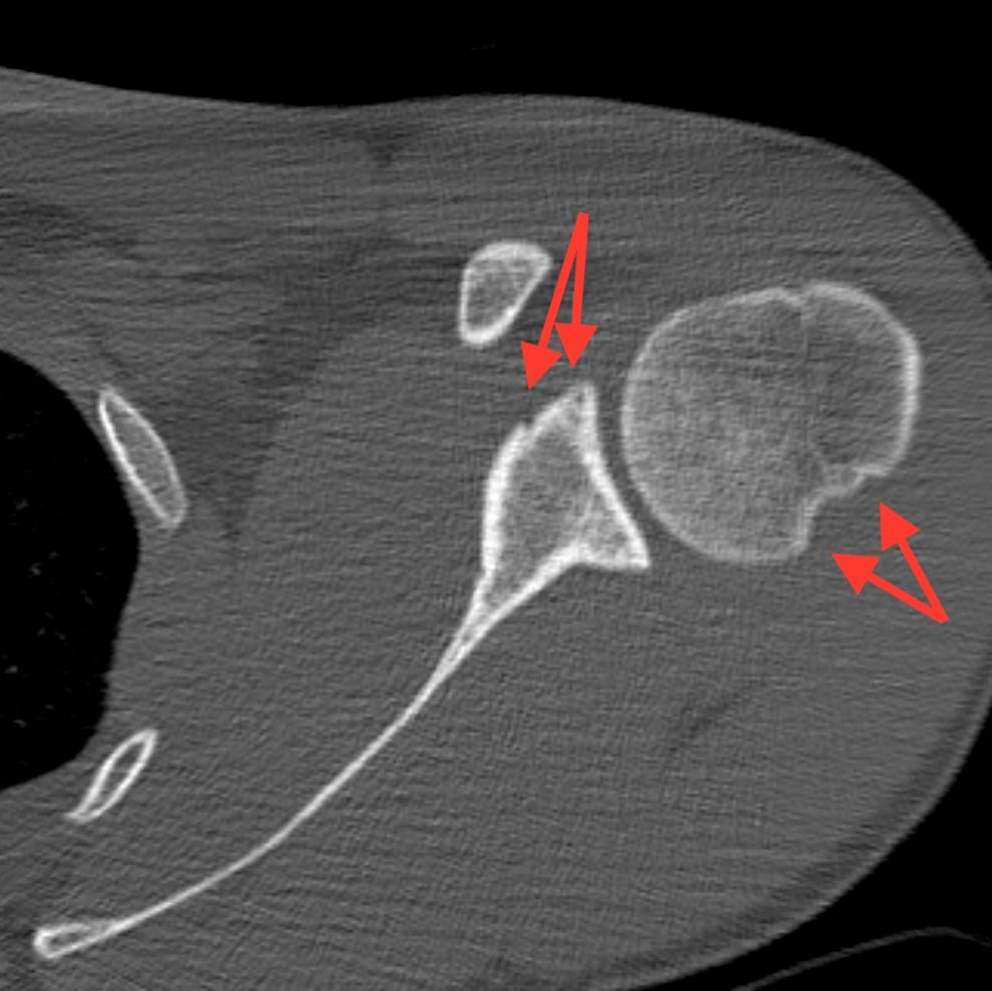
Computed tomography scan of the left shoulder, axial cross-sectional image, demonstrates mild compression, or Bankart lesion, of the anterior glenoid fossa over a 1.1-cm area (upper arrows, anterior) and a Hill-Sachs lesion measuring 1.4 cm × 0.3 cm on the humeral head (lower arrows, posterior).

**Figure 3.**
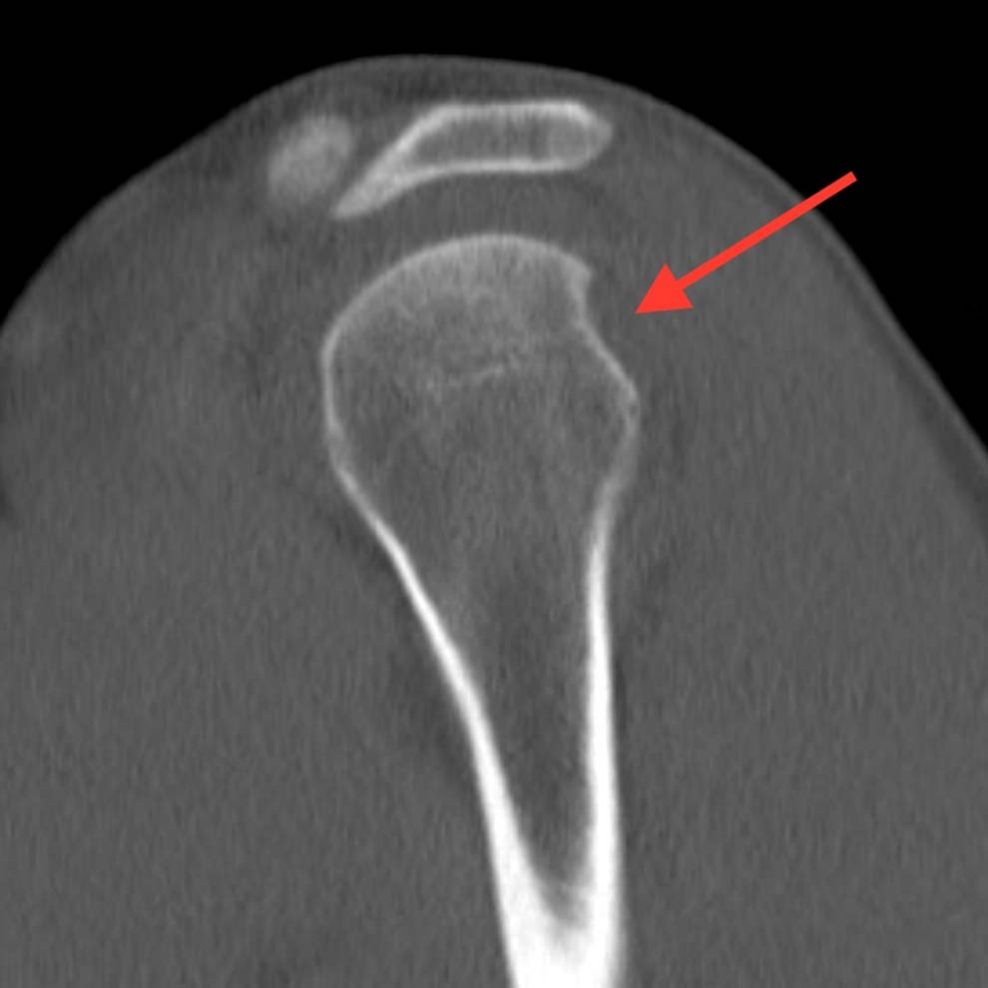
Computed tomography scan of the left shoulder, sagittal cross-sectional image, shows the Hill-Sachs lesion on the posterior humeral head (arrow).

**Figure 4.**
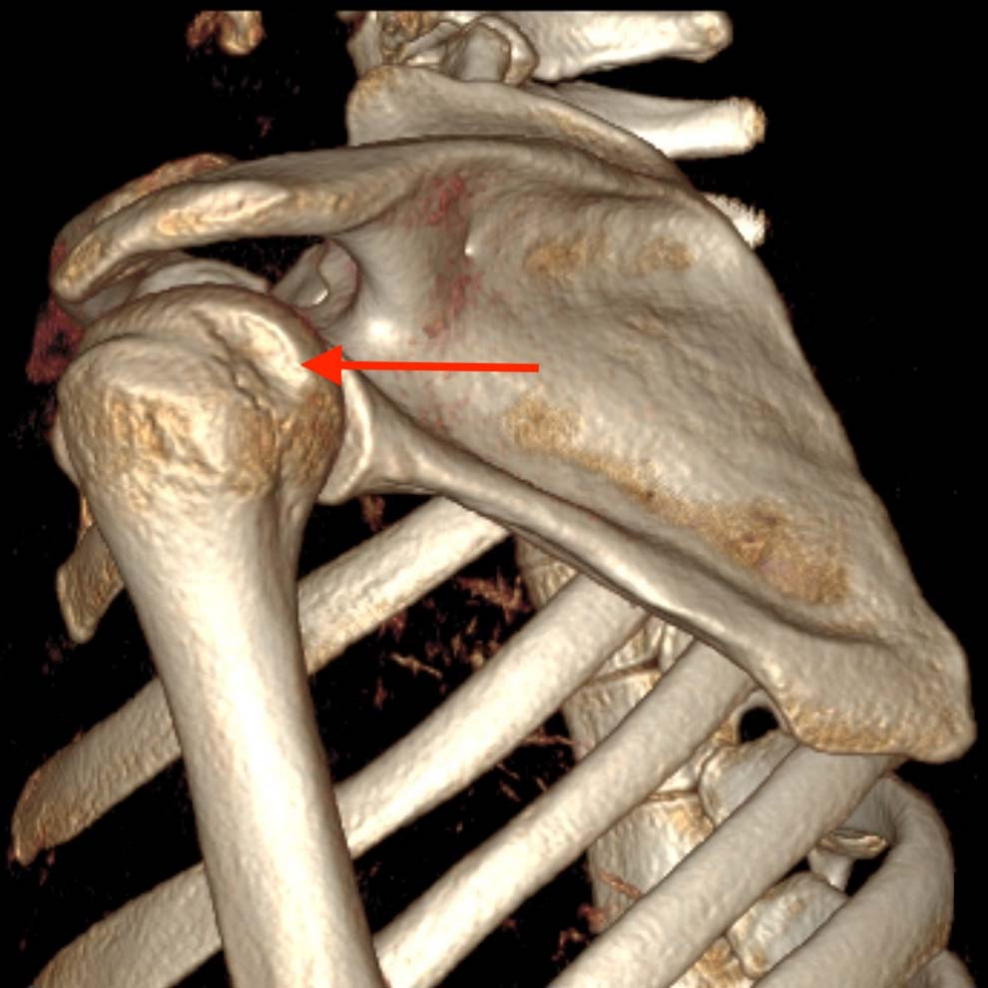
Three-dimensional reconstruction of the left shoulder shows the Hill-Sachs lesion on the posterior humeral head (arrow).

Closed 3 Tesla magnetic resonance imaging of the patient's left shoulder without contrast completed 5 days after the orthopedic evaluation showed a 270° labral tear, including a superior labrum anterior-to-posterior lesion extending into the biceps anchor, an anterior inferior glenoid Bankart labral tear with capsular injury, and a posterior inferior labral tear. Only the posterior superior labrum remained attached to the glenoid. A type II to type III acromion was also visualized, with associated rotator cuff tendinosis. Noncontrast computed tomography scan of the left shoulder showed mild compression of the anterior glenoid fossa with preservation of the glenoid surface area over a 1.1-cm area (Figure 2). A Hill-Sachs deformity measuring 1.4 cm × 0.3 cm was visualized (Figures 2, 3, and 4).

The patient was scheduled to undergo arthroscopic left glenoid labrum repair and remplissage procedure to treat his recurrent shoulder dislocations and associated structural injuries. The patient played soccer 2 days before his scheduled surgery and had another dislocation. The shoulder remained dislocated for several hours until it was reduced in the emergency department, potentially enlarging the Hill-Sachs lesion.

**Figure 5.**
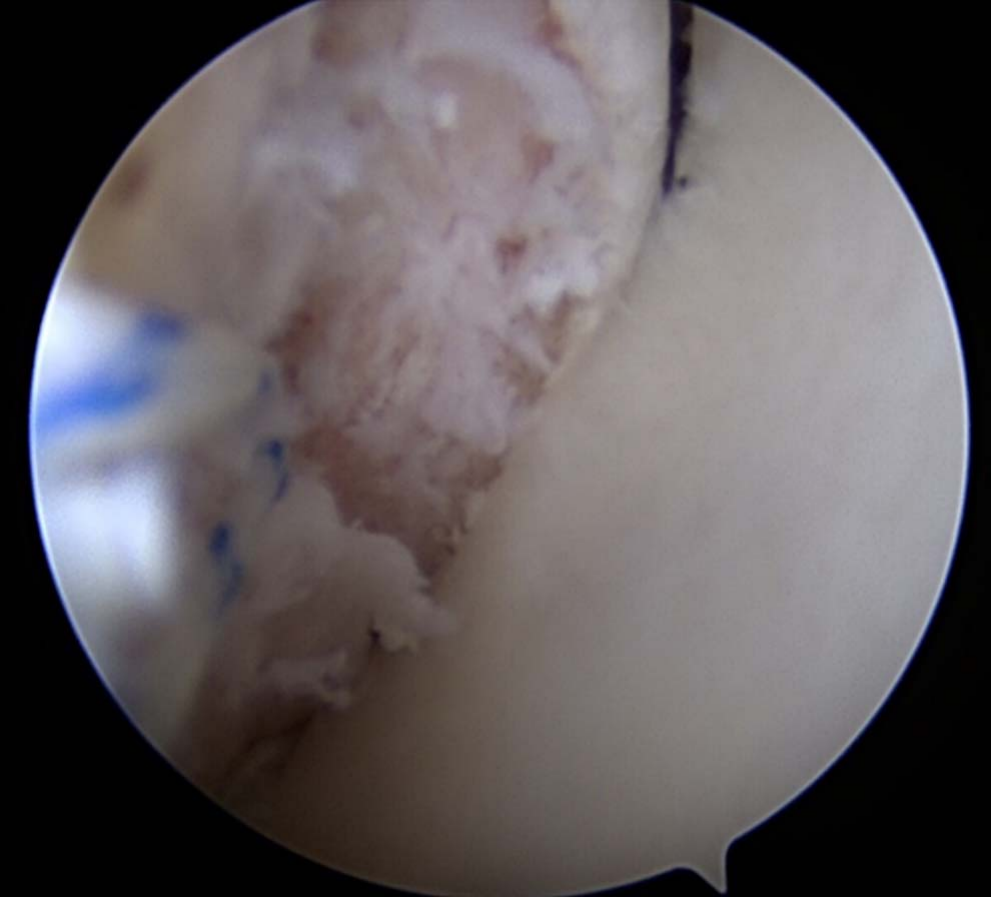
Arthroscopic image within the shoulder joint shows the humeral head dislocating while in 30° of forward flexion and neutral rotation.

**Figure 6.**
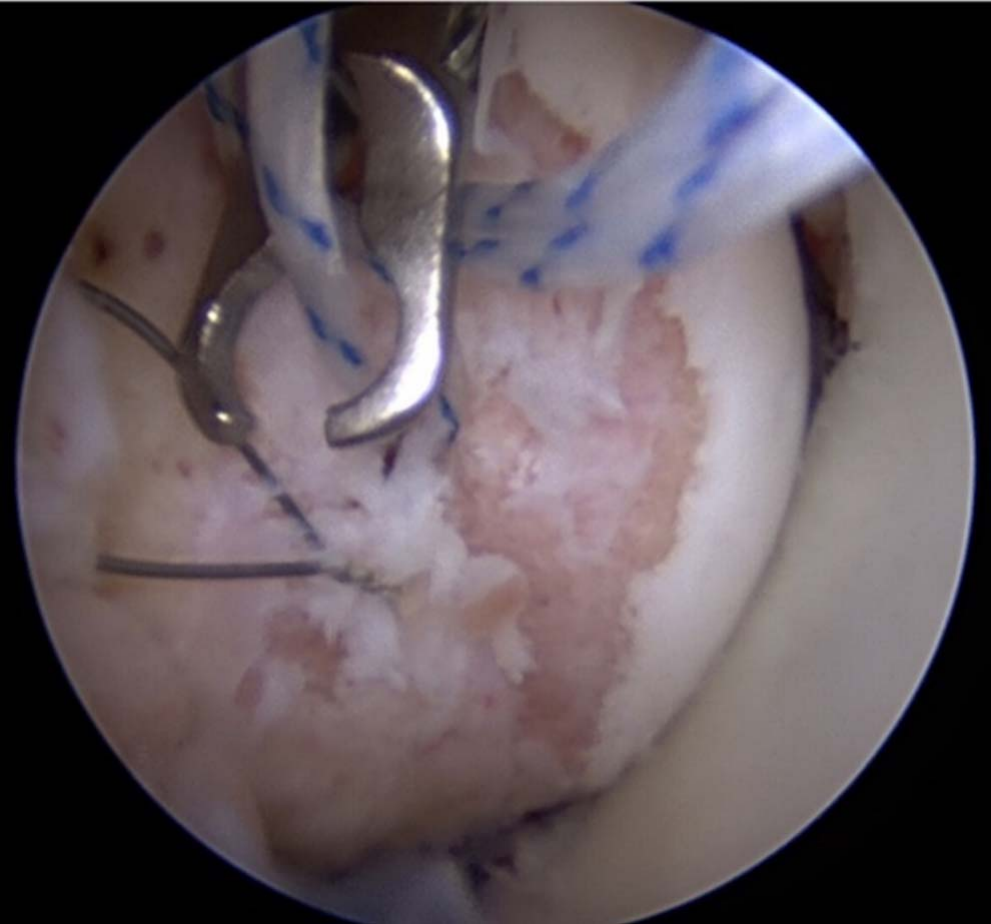
Arthroscopic image within the shoulder joint shows the humeral head perched and a large Hill-Sachs lesion. A percutaneous suture passer is placing suture in the Hill-Sachs lesion.

Surgical intervention with the patient under general anesthesia included a remplissage procedure, repair of the glenoid labrum, capsular plication, debridement, and acromioplasty. The shoulder demonstrated significant instability, dislocating with anteriorly directed force while in the neutral position. Forward flexion to 30° in neutral rotation also caused the shoulder to dislocate (Figure 5). Diagnostic arthroscopy demonstrated a 270° labral tear with only the posterior labrum intact, as well as a large and bleeding Hill-Sachs lesion. This lesion engaged in 45° of flexion and abduction and 60° of external rotation. The Hill-Sachs lesion, multiple loose bodies, free-floating cartilage, and devitalized tissue were debrided. Six anchors were placed in the glenoid for a pan-labral repair and capsular shift. The remplissage sutures were tied in pairs, reducing the infraspinatus into the Hill-Sachs defect. The tails of the paired anchors were tied to each other, creating a compression grid across the infraspinatus above the Hill-Sachs defect (Figure 6).

The patient was placed in a sling with an abduction pillow for 6 weeks. Two weeks postoperatively, he began shoulder pulley exercises, and 3 weeks postoperatively, he began formal physical therapy. Six weeks postoperatively, the patient had 130° of forward flexion, 130° of abduction, and 20° of external rotation. Twelve weeks postoperatively, the patient began a gradual strengthening program. At 7-month follow-up, the patient had full range of motion and was no longer in formal physical therapy. He had returned to soccer and running without pain or instability. He did not require long-term medical management or recommendations.

## DISCUSSION

Understanding the risks involved with indoor skydiving is important, especially with expansion of the sport and the campaign to add it to the 2024 Olympic Games.^7^ The airstream force and body position of indoor skydiving have the potential to dislocate the shoulder. This mechanism has been studied and described in outdoor skydiving. In a 2007 analysis of outdoor jumps in Sweden, 3 of 7 injuries sustained during free fall were shoulder dislocations caused by the force of the airstream, with an additional 7 dislocations occurring during the rest of the jump.^4^ However, shoulder injuries accounted for only 3.9% to 4.8% of the total outdoor skydiving injuries documented in Sweden between 1999 and 2003 and at the 2000 and 2001 World Freefall Convention in Illinois.^4,9^ This rate of shoulder injury may increase dramatically when the sport is brought indoors, with one author indicating that 51% of reported injuries involved the shoulder joint.^8^ These data suggest that the indoor variant of skydiving may provide additional risk of traumatic shoulder injury or dislocation compared to outdoor skydiving.

Hill-Sachs lesions are distinct osseous defects in the head of the humerus and are typically caused by traumatic anterior shoulder dislocations.^6^ The relatively soft posterolateral aspect of the humeral head forcefully collides with the harder anterior bone of the glenoid rim, leading to the characteristic posterolateral humeral compression fracture known as a Hill-Sachs lesion.^10^ Of patients with chronic anterior shoulder instability, 93.3% were found to have Hill-Sachs lesions upon arthroscopic investigation.^11^

In our case, the severe instability of the patient's shoulder, notable bone loss, and recurring nature of his dislocations indicated that arthroscopic labral repair and capsular plication alone would have been insufficient in resolving his symptoms.^12^ Both a coracoid bone transfer to the glenoid (Latarjet procedure) and remplissage procedure were considered in addition to labral repair.^13,14^ While both the Latarjet and remplissage procedures have comparable clinical outcomes, the remplissage procedure has a lower complication rate in the setting of subcritical glenoid bone loss. The specific technique used in our case is similar to that described by Purchase et al, using an arthroscopic remplissage and Bankart to stabilize a shoulder with glenoid bone loss and a large Hill-Sachs lesion.^15^

This case elucidates a previously undescribed mechanism and setting of shoulder dislocation, in which the participant is already in the vulnerable abducted and externally rotated position in the wind tunnel and reaches for the stationary tunnel wall, forcing the arm further into external rotation and dislocating the shoulder. As indoor skydiving becomes more accessible and popular, physicians and participants alike must understand the risks, particularly when shoulder dislocations may have a disproportionately increased prevalence compared to other injuries such as neck strain or knee injuries. While the injury-per-jump numbers of outdoor skydiving are known (ranging from 48 injuries per 100,000 jumps to 174 injuries per 100,000 jumps), the injury rate for indoor skydiving is not well established.^4,9^ Studies of injuries associated with indoor skydiving are an important next step in evaluating the safety of the sport.

## CONCLUSION

Indoor skydiving may predispose participants to anterior dislocations because an already-abducted shoulder can be forced into hyper-external rotation. In this case, we presented the mechanism and setting of anterior shoulder dislocation that was treated arthroscopically with labral repair, capsular plication, and remplissage procedure. The patient made a complete recovery from a debilitating injury without any loss of range of motion. We advocate consideration of the remplissage procedure for engaging Hill-Sachs lesions and advise caution before participation in indoor skydiving by any individual, but especially those with a history of shoulder instability.

## Notes

*This article meets the Accreditation Council for Graduate Medical Education and the American Board of Medical Specialties Maintenance of Certification competencies for Patient Care and Medical Knowledge.*

## References

[1] Dwyer T, Petrera M, Bleakney R, Theodoropoulos JS. Shoulder instability in ice hockey players: incidence, mechanism, and MRI findings. Clin Sports Med. 2013;32(4):803-813. doi: 10.1016/j.csm.2013.07.013 24079436

[2] Shah AA, Selesnick FH. Traumatic shoulder dislocation with combined Bankart lesion and humeral avulsion of the glenohumeral ligament in a professional basketball player: three-year follow-up of surgical stabilization. Arthroscopy. 2010;26(10):1404-1408. doi: 10.1016/j.arthro.2010.03.018 20887939

[3] Owens BD, Duffey ML, Nelson BJ, DeBerardino TM, Taylor DC, Mountcastle SB. The incidence and characteristics of shoulder instability at the United States Military Academy. Am J Sports Med. 2007;35(7):1168-1173. doi: 10.1177/0363546506295179 17581976

[4] Westman A, Björnstig U. Injuries in Swedish skydiving. Br J Sports Med. 2007;41(6):356-364. doi: 10.1136/bjsm.2006.031682 17224436 PMC2465315

[5] Westman A. Shoulder injuries have been noted as a recurring problem in skydiving. J Trauma. 2005;59(4):1033. doi: 10.1097/01.ta.0000188014.44804.da 16374303

[6] Bost FC, Inman VT. The pathological changes in recurrent dislocation of the shoulder: a report of Bankart's operative procedure. J Bone Joint Surg. 1942;24(3):595-613.

[7] Olympic Games, from dream to reality. #Flyin2024. Accessed April 25, 2021. www.flyin2024.com/en/olympic-games-from-dream-to-reality

[8] Rodrigues A. All pain, no plane: my grim experience with indoor skydiving. Medium.com. December 7, 2018. Accessed January 10, 2019. medium.com/s/story/all-pain-no-plane-my-experience-with-indoor-skydiving-ddc2b0c1f94a

[9] Barrows TH, Mills TJ, Kassing SD. The epidemiology of skydiving injuries: World Freefall Convention, 2000-2001. J Emerg Med. 2005;28(1):63-68. doi: 10.1016/j.jemermed.2004.07.008 15657007

[10] Hill HA, Sachs MD. The grooved defect of the humeral head. A frequently unrecognized complication of dislocations of the shoulder joint. Radiology. 1940;35(6):690-700. doi: 10.1148/35.6.690

[11] Yiannakopoulos CK, Mataragas E, Antonogiannakis E. A comparison of the spectrum of intra-articular lesions in acute and chronic anterior shoulder instability. Arthroscopy. 2007;23(9):985-990. doi: 10.1016/j.arthro.2007.05.009 17868838

[12] Giles JW, Elkinson I, Ferreira LM, Moderate to large engaging Hill-Sachs defects: an in vitro biomechanical comparison of the remplissage procedure, allograft humeral head reconstruction, and partial resurfacing arthroplasty. J Shoulder Elbow Surg. 2012;21(9):1142-1151. doi: 10.1016/j.jse.2011.07.017 22036545

[13] Yang JS, Mehran N, Mazzocca AD, Pearl ML, Chen VW, Arciero RA. Remplissage versus modified Latarjet for off-track Hill-Sachs lesions with subcritical glenoid bone loss. Am J Sports Med. 2018;46(8):1885-1891. doi: 10.1177/0363546518767850 29672132

[14] Cho NS, Yoo JH, Rhee YG. Management of an engaging Hill-Sachs lesion: arthroscopic remplissage with Bankart repair versus Latarjet procedure. Knee Surg Sports Traumatol Arthrosc. 2016;24(12):3793-3800. doi: 10.1007/s00167-015-3666-9 26044354

[15] Purchase RJ, Wolf EM, Hobgood ER, Pollock ME, Smalley CC. Hill-Sachs "remplissage": an arthroscopic solution for the engaging Hill-Sachs lesion. Arthroscopy. 2008;24(6):723-726. doi: 10.1016/j.arthro.2008.03.015 18514117

